# A high-frequency sense list

**DOI:** 10.3389/fpsyg.2024.1430060

**Published:** 2024-08-09

**Authors:** Lei Liu, Tongxi Gong, Jianjun Shi, Yi Guo

**Affiliations:** ^1^English Department, School of International Studies, Zhengzhou University, Zhengzhou, China; ^2^Institute of Linguistics, Shanghai International Studies University, Shanghai, China; ^3^Department of College English, School of Languages, Shanghai University of International Business and Economics, Shanghai, China

**Keywords:** BERT, semantic annotation, sense frequency, word list, large language model

## Abstract

A number of high-frequency word lists have been created to help foreign language learners master English vocabulary. These word lists, despite their widespread use, did not take word meaning into consideration. Foreign language learners are unclear on which meanings they should focus on first. To address this issue, we semantically annotated the Corpus of Contemporary American English (COCA) and the British National Corpus (BNC) with high accuracy using a BERT model. From these annotated corpora, we calculated the semantic frequency of different senses and filtered out 5000 senses to create a High-frequency Sense List. Subsequently, we checked the validity of this list and compared it with established influential word lists. This list exhibits three notable characteristics. First, it achieves stable coverage in different corpora. Second, it identifies high-frequency items with greater accuracy. It achieves comparable coverage with lists like GSL, NGSL, and New-GSL but with significantly fewer items. Especially, it includes everyday words that used to fall off high-frequency lists without requiring manual adjustments. Third, it describes clearly which senses are most frequently used and therefore should be focused on by beginning learners. This study represents a pioneering effort in semantic annotation of large corpora and the creation of a word list based on semantic frequency.

## 1 Introduction

Learning vocabulary is essential for foreign language learners. Beginning learners often face the challenge of determining which words to learn first. Frequency has proven a useful tool in selecting the important words to prioritize and “frequency lists have been used for decades for teaching the most useful general words” (Vilkaite-Lozdiene and Schmitt, 2020, p. 81). Such high-frequency word lists are especially needed for foreign language learners as they have limited exposure to the target language and therefore cannot acquire high-frequency words as native speakers do.

A number of high-frequency word lists have been created to help foreign language learners master English vocabulary. Influential word ones include West's (1953) General Service List (GSL), Nation's (2012) BNC/COCA list, Browne et al.'s (2013) NGSL and Brezina and Gablasova's (2015) New-GSL.^1^ It is believed that by using high-frequency word lists, beginning learners can get the best return for their learning efforts (Nation, 2013; Dang et al., 2022). These word lists become important references for classroom teaching, vocabulary assessments, and textbook compiling. Several studies have confirmed the usefulness of these word lists both in terms of coverage and teachers' perceptions. Nakayama (2022) found the NGSL gave between 92.8 and 95.8% coverage for the three top-selling MEXT-approved high school textbooks.^2^ Dang et al. (2022) reported the usefulness of BNC/COCA 2000 in teachers' perception.

These word lists, despite their widespread use, did not take word meaning into consideration.

First, most word lists simply catalog the spelling forms of high-frequency words without explaining their meanings. This seems to imply that the meaning is not important or that all meanings are equally important. When learners consult these word lists, they do not know which meaning(s) to prioritize. Szudarski (2018) raised the question of whether beginning learners should focus only on the most frequent meaning or on all the meanings of these listed words. For example, “act” is usually identified as a high-frequency word in previous word lists. Should a middle school student in China study all the meanings of this word or only focus on the most frequent ones? Szudarski's doubt invites another question. If learners want to focus on the frequent meanings first, what are the most frequent meanings–those related to “behave,” “perform,” or “written law”? Foreign language learners, even foreign language teachers, are not always clear about such questions. Dictionaries do not provide detailed information on semantic frequency either. Most dictionaries claim that different meanings of a word are usually given in order of frequency, with the most frequent meaning given first. However, a comparison reveals that different dictionaries display senses in quite different orders.

Another issue with earlier word lists was their inability to accurately identify high-frequency terms. Previous high-frequency lists use word family, lemma or flemma as their counting unit. A word family consists of a base word and all its derived and inflected forms that can be understood by a learner without having to learn each form separately (Bauer and Nation, 1993). A lemma is a word family where the family members consist of the headword and inflected forms of the same part of speech (Bauer and Nation, 1993). A flemma is a word family that consists of a headword and inflected forms of different parts of speech (Pinchbeck, 2014). Typically flemmas include more members than lemmas. Word lists based on word families tend to include many low-frequency words. For example, GSL includes such words as “particle” or “unpleasantly.” As pointed out by Brezina and Gablasova (2015), these words are included not because they are highly frequent, but because they belong to the same family of high-frequency items such as “part” and “please.” Such less frequent members, Nation (1990) pointed out, will increase students' learning burden. On the other hand, previous word lists overlooked many common everyday words such as “Monday” and “April.” When compiling the BNC/COCA list, Nation had to manually include 186 word families of low frequency into high-frequency bands. Such words include days of the week (e.g., “Monday,” “Friday”), months of the year (e.g., “April,” “July”), and children's language (e.g., “naughty,” “silly,” “rabbit,” and “orange”). Nation made such an adjustment because these words cannot be included in the high-frequency band solely based on frequency (Nation, 2016). Similarly, NGSL consists of 52 words for which frequency ranking information is not available. They include days of the week, months of the year and numerical words.^3^ In short, previous lists based on word frequency included some less frequent words while at the same time tending to omit some common words if without manual adjustment.

The root of these problems lies in the conventional definition of “word” which is based solely on its spelling form but neglects the different senses that each form can represent. For example, different forms of “act” are counted as one single word, regardless of their specific senses. These senses are different, especially in the eyes of foreign language learners. Bogaards (2001, p. 324) noted that “the only reason to lump together all these semantically disparate elements in the category of words seems to be the fact that they constitute groups of letters between two blanks.” This form-based definition of “word” brings problems in creating word lists based on frequency. It implies that a word with multiple meanings (such as “act”) is more likely to be categorized as high-frequency compared to a word with only one meaning (like “Monday”).

Previous studies (Gardner, 2007; Gardner and Davies, 2007) proposed to solve this problem by redefining “word” as a union of one single form and one single meaning. That is to say, different senses of “act”—“act^1^ (perform),” “act^2^ (behave),” or “act^3^ (written law)”—will be treated as different items. By so doing, we can solve the two problems raised above. First, we can identify which sense of a word is most frequently used, and therefore provide better reference for learners consulting word lists. Second, when we use sense instead of word as the counting unit, a single-meaning word like “Monday” is no longer in competition with a lump of all the meaning representations of “act,” but with “act^1^,” “act^2^,” or “act^3^,” respectively. Consequently, “Monday” might be able to get into the high-frequency band without necessitating manual adjustments as in Nation's case.

No attempts have been made to carry out such proposed analysis. Gardner (2007) recognized that it would be virtually impossible to meet the criteria of the same meaning in grouping words unless corpora are semantically tagged. However, corpora of this nature are “in their developmental infancy” (Gardner, 2007, p. 244), or in other terms, “are much easier talked about than constructed” (Gardner and Davies, 2007, p. 353).

The daunting, almost insurmountable task of semantically annotating a large corpus has now become achievable with the recent advancement of large language models. Google's *Bidirectional Encoder Representations from Transformers* (BERT) can now represent each word in a corpus using a 1024-dimensional vector. A vector can be simply represented as a number list or array. Different meanings of a word will be represented by distinct vectors and subsequently be treated separately.

In this article, we will annotate COCA and BNC semantically with a BERT model, count different senses, calculate semantic frequency, create a high-frequency sense list, check its validity, and finally discuss methodological and pedagogical implications.

## 2 Research questions, data, and tool

### 2.1 Research questions

The objective of this study is to identify the most frequently used senses of English words, with a particular focus on addressing the following key questions. First, what are the high-frequency senses in English? Second, are these high-frequency senses selected from COCA representative of other English varieties? Third, what are the pedagogical and methodological implications the study can bring?

### 2.2 Sense inventory

In this study, “sense” refers to sense entries listed in the Oxford English Dictionary (OED).^4^

There is considerable controversy about what constitutes a sense and how senses are distinguished from one another. Atkins (1991) noted that lexicographers could variously lump senses together, split them apart, or combine elements of meaning in different ways. This point can be easily illustrated when we compare the entries of the same word in different dictionaries.

In this study, we choose to use OED for three reasons. First, a result based on an English dictionary is more suitable for foreign language learning purposes. Natural language processing studies often use WordNet as their sense inventory. WordNet is constructed around sense relationships and its sense definitions are not suitable to be used in foreign language classrooms. Second, OED provides more extensive example sentences compared to other available dictionaries. This, as our preliminary study shows, can improve the accuracy of sense annotation using a BERT model. Third, it provides a general-level sense and a more specific sub-sense. In OED, sense entries are organized into two levels: general senses and sub-senses. The boundary between two general-level senses is usually clear. Some other dictionaries adopt a single-level organization, in which case, boundaries between some senses are not clear-cut. Computer models have difficulty distinguishing fine-grained senses. Previous studies (Edmonds and Kilgarriff, 2002; Ide and Wilks, 2007) have shown that even human annotators could not distinguish well between fine-grained senses. To improve annotation accuracy, we use the general-level sense in OED.

Phrasal verbs are treated as unique sense units.^5^ Previous research (Yuan et al., 2016; Hu et al., 2019) assigned all occurrences of a word in a corpus to sense entries in a dictionary. However, this approach inevitably leads to some inaccuracies. Phrasal verbs, such as “give up,” have unique meanings as a whole and cannot be classified under any “give” or “up” sense entry. To deal with this issue, we decide to treat phrasal verbs as unique items. There is no consensus about what a phrasal verb is. Different linguistic theories have different definitions and different dictionaries list different entries. For easier operation, we include only the phrasal verbs listed in OED.

To sum up, different sense entries as well as the entries for phrasal verbs in OED will be counted.

### 2.3 Corpora

Three corpora are used in this study. The Corpus of Contemporary American English (COCA) is used to calculate the sense frequencies of English words. The British National Corpus (BNC) is used to check how much the lexical characteristics of high-frequency senses in COCA can represent both American English and British English.

COCA is a large and balanced corpus of contemporary American English. According to its official introduction, it contains more than one billion words of text from eight genres.^6^ COCA is available in several versions, namely DB (suitable for databases), WLP (one word per line), and TXT (the most common text format). We have chosen the TXT version.

BNC is a large collection of over 100 million words of written and spoken language, which were gathered from a wide array of sources to provide an accurate reflection of British English during the latter half of the twentieth century. It has been a source to make frequency lists in a number of vocabulary studies. Several versions of BNC are now available. In this study, the XML edition^7^ is used.

It is recognized that there are distinct time frames covered by these two corpora—with COCA spanning materials from 1990 onwards, while BNC focuses on materials in the 1990's. Ideally, a more current corpus of British English such as “BNC 2014” would be preferred. However, access to “BNC 2014” is currently limited to specific web interfaces or software such as LancsBox. This restriction impedes the ability to analyze the entire text using BERT on a local computer. Other contemporary British English corpora such as the Brown family or the LOB family corpora are generally not large enough to produce reliable results, making BNC the 2nd best choice for this study.

### 2.4 Tool

Google has open-sourced multiple BERT models (Devlin et al., 2018). The model used in this study is “wwm_uncased_L-24_H-1024_A-16,”^8^ the largest model released by Google, which consists of 24 layers, 1,024 hidden units and 16 self-attention heads with 340 million parameters. According to the evaluation results on multiple natural language processing tasks released by Google for different versions of models, generally, the larger the model parameters, the better the performance. For this reason, our study has chosen the largest version of BERT.

During the pre-training phase, BERT utilizes two tasks: the Masked Language Model (MLM) and the Next Sentence Prediction (NSP). The MLM task is similar to fill-in-the-blanks on a test; given a sentence, one or more words are randomly masked, and the model must predict the masked words based on the remaining vocabulary. The NSP task involves determining whether the second sentence in a given pair from the same corpus is the sequential follow-up to the first sentence, a binary classification task with only “yes” or “no” as answers. This NSP task is akin to paragraph-ordering questions seen in exams. It is evident that the pre-training process of the BERT model is similar to the human language learning process; the MLM task allows the model to learn vocabulary-related knowledge, while the NSP task enables the model to acquire semantic information at the sentence and even paragraph levels. Combining these two tasks allows the model to produce word vectors that comprehensively and profoundly depict the information in the input text. Essentially, the pre-training process involves continuous adjustment of the model parameters to enhance the model's understanding of language.

The pre-trained model released by Google is based on the English Wikipedia corpus. Although the Wikipedia corpus is extensive in scale, it contains many non-standard languages. To enhance the model's semantic differentiation accuracy, we fine-tuned the model using the entire corpus COCA, BNC, and the Corpus of Historical American English (COHA).

## 3 Methods and procedures

In this article, we annotate COCA semantically by using BERT, a deep-learning language model developed by Google. After the corpora are semantically annotated, we calculate the frequency of each sense and select high-frequency senses. We then check the coverage of these high-frequency senses and their validity. Finally, we discuss methodological and pedagogical implications.

### 3.1 Data cleaning

The data cleaning in this article mainly involves deleting incomplete sentences from the COCA corpus, as well as tokenization, lemmatization, and part-of-speech tagging.

For the COCA corpus, we used Spacy to segment the text into sentences and then filtered out invalid sentences containing the “@” symbol. Due to copyright reasons, ~5% of the content in the COCA corpus sold by Davies has been randomly replaced with “@” symbols, meaning that every 200 words will have 10 words deleted and replaced with “@.” We completely removed such sentences. For the valid sentences remaining, we used Spacy for tokenization, lemmatization, and part-of-speech tagging. After removing these fragmented sentences, the size of COCA used in this study is ~783 million words.

The processing of the BNC data is relatively straightforward. We followed the original annotation scheme of the corpus and extracted four core fields: word, lemma, part of speech, and text type.

### 3.2 Semantic annotation

The semantic annotation of a large corpus consists of the following steps.

First, given a word *w* with *m* senses, for the *i*_*th*_∈{1, 2, …, *m*} sense, it has *n*_*i*_ example sentences. For the *j*_*th*_∈{1, 2, …, *n*_*i*_} example sentence containing the target word *w* with the *i*_*th*_ sense, we extract the token vector of the target word *w*, denoted as *e*_*ij*_∈ℝ. The word vector *e*_*i*_ of the *i*_*th*_ sense can be obtained by averaging the token vectors of the target word in the example sentences, i.e., *e*_*i*_ = mean(*e*_*i*1_, *e*_*i*2_, …, *e*_*i*_*n*__*i*__). Finally, we obtain the word vectors *e*_1_, *e*_2_, …, *e*_*m*_ of the *m* senses of word *w*, which serve as the sense representation for each sense.

We use a Python program to access Oxford Dictionary API and get an entry in OED. An entry consists of all definitions and example sentences. Then, example sentences for each definition from OED are fed into BERT. BERT analyzes these sentences and represents each definition with a 1024-dimensional vector. As a result, we get a vector for each definition of every entry from OED.

This representation process can be illustrated with a simple example. The word “capital” has eight senses in OED,^9^ comprising four nouns, three adjectives, one exclamation. The first noun sense refers to the “city” sense. This sense entry contains 40 example sentences in total. Of these sentences, 28 (70%) example sentences are fed into the BERT model. BERT calculates the vector of this sense from each of the 28 example sentences, computes the average value, and finally represents it with a 1024-dimensional vector. By repeating this process, we get a vector for each of the remaining seven senses of the word “capital.”

Second, each sentence in the corpus is analyzed by BERT, and similarly, each word is represented with a vector.

Next, for a sentence containing the target word *w* extracted from COCA, we first obtain the token representation *e*_*t*_ for the target word. Then, we calculate the similarity between *e*_*t*_ and *m* sense representations of the target word *m*. The sense with the highest similarity score is selected as the sense of the target word in the sentence. There are many methods to calculate similarity, and this study adopts the most commonly used cosine similarity algorithm. The sense of the target word in a sentence can be formally expressed as:


$$i^{*}=\underset{i\in 1,2,\ldots ,m}{\text{arg max}}\cos\text{\_}sim\left(e_{t},e_{i}\right)$$

To put it another way, every vector for each word in the corpus (obtained in step 1) is compared with the vectors derived from definitions in OED (obtained in step 2). The similarity between the vectors is assessed. The definition with the highest similarity score is selected as the meaning of the target word.

For instance, let's consider the semantic annotation of the word “capital” in sentence ① in COCA.

① The UN escorted buses carrying more than 300 mothers and children out of the Bosnian capital Sarajevo yesterday.

**Figure 1:**
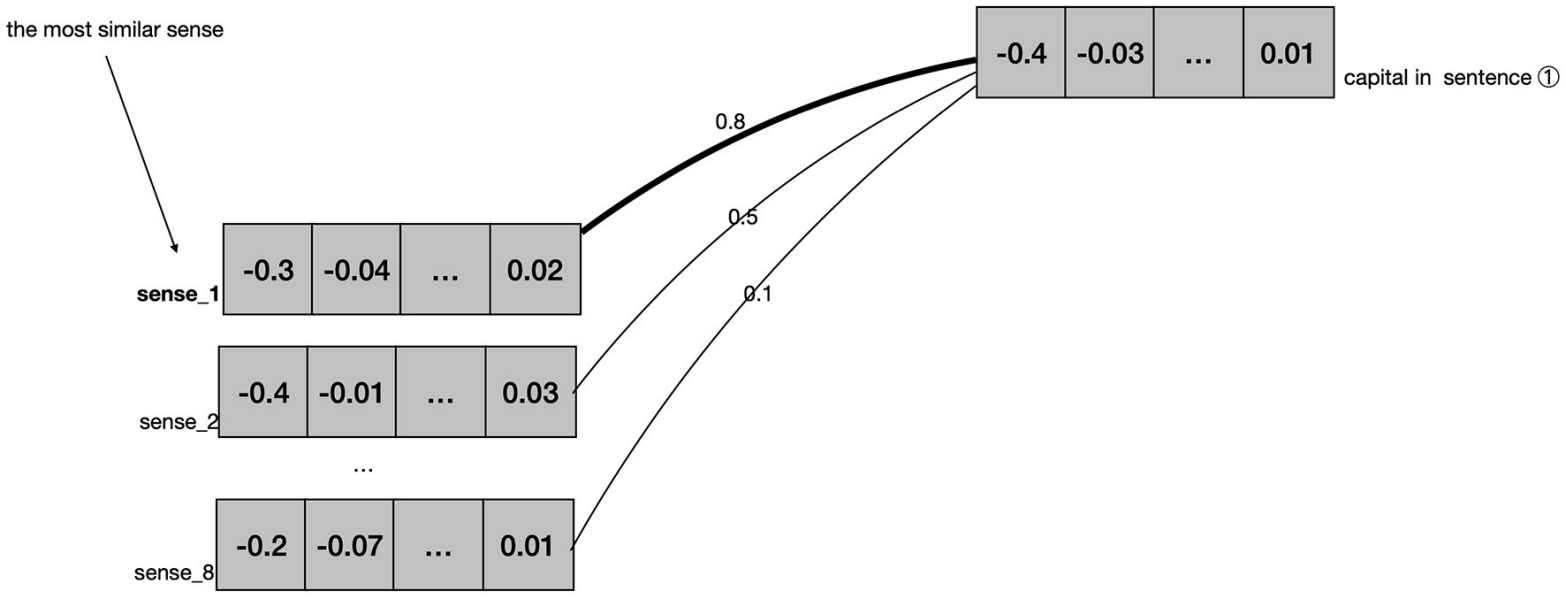
Semantic annotation of “capital” in sentence ①.

The word “capital” has 8 senses in OED. In the first step, BERT will analyze the example sentences of the word “capital” in OED, and represent each of the eight senses with a vector, as shown in the left part of Figure 1. In the second step, BERT will analyze every sentence that contains the word “capital” in COCA. When BERT meets sentence ① in COCA, it will generate a 1024-dimensional vector to represent the word “capital,” as shown in the right part of Figure 1. After that, BERT will compare this vector representing “capital” in sentence ① with each vector representation of the different sense entries for “capital” in OED, selecting the most similar vector as the definition for “capital” in this specific sentence. In this instance, the word “capital” in sentence ① is found to be most similar to the first sense listed in the OED. Therefore, it is annotated with the first sense.

These steps are repeated for every word in the corpus and consequently, each word in the corpus is matched with a definition in OED. The entire corpus is then semantically annotated.

Phrasal verbs have their meanings as a whole and are analyzed as unique sense units in this study. Regrettably, the BERT model cannot calculate vectors for phrases satisfactorily, phrasal verbs are therefore identified by rules in this study.

We specify four rules to single out phrasal verbs in corpora. First, the first element in a phrasal verb must be a verb, and the second element must be an adverbial particle. Second, at most two words can be inserted between the verb and adverbial particle. Third, if a phrasal verb is made up of three words, the first element must be a verb, the second one must be an adverbial particle and the third must be a preposition. Fourth, phrasal verbs are identified by maximum string matching.

The first two rules are similar to the rules specified in Gardner and Davies (2007). These two rules will rule out false phrasal verbs “take in” in sentence ② and include non-continuous examples such as “look sth up” in sentence ③.

② The direction the English novel was to **take in** the post-war era was the subject of much discussion in the late 1950s
③ If I want information I can **look it up** and read it far faster than I can get it from a screen.

Gardner and Davies (2007) assume that all phrasal verbs are made up of two items. When studying sentences from COCA, we find that some phrasal verbs consist of three items, such as “look up to” and “look forward to.” We have to make two more rules to include such three-word phrasal verbs.

During the whole annotation process, if a word is part of a phrasal verb, BERT will not associate any specific definition with it. For instance, the phrase “look up to” will be considered as a single entity. The individual words “look,” “up,” or “to” will not be linked to any particular sense entry in OED.

### 3.3 Accuracy check

During the annotation process, the example sentences from the OED are divided into two groups: a training group and a test group. The training group, consisting of 70% of the example sentences, is inputted into BERT to generate vectors for definitions sourced from the OED. The test group, accounting for 30% of the example sentences, is combined with sentences from the COCA. When BERT analyzes sentences in the second step specified in Section 3.2, it does not differentiate whether a sentence is an example sentence for a definition in OED or a random sentence in COCA. After the annotation, the example sentences are singled out, and their annotations are compared with their original definitions. The accuracy of the annotations is determined by calculating the number of words that have been correctly annotated.

### 3.4 Selection of high-frequency sense

Different studies have adopted varying criteria for developing high-frequency word lists. West (1953) used both subjective and objective criteria. Brezina and Gablasova (2015) took a purely quantitative approach. Nation (2012) used frequency as the primary criterion but made some manual adjustments. To ensure replicability, we have predominantly adopted a quantitative approach. While some subjective judgments have been made, they are also conducted automatically and can be replicated easily.

A high-frequency sense list is created according to sense frequency, style neutrality, and range.

First, all semantically annotated words in COCA are ranked in descending order based on their frequency. After COCA is semantically annotated, words of different senses are treated as distinct units. That is to say, the occurrences of “act^1^ (perform),” “act^2^ (behave),” and “act^3^ (a written law)” are not collectively counted as instances of one word “act.” Instead, the occurrence of these different senses is counted individually. All these senses are ranked according to their frequency. Frequency has always been the most important standard in the development of word lists. Similarly, it is used as the first standard in this study.

Second, any senses that possess a register label, are limited to specific domains, or are proper nouns, or are predominantly used in regions outside of the UK or the US, are eliminated. According to West (1953), foreign learners at the early stages of language acquisition should first acquire stylistically or emotionally neutral terms. As such, senses labeled as “formal,” “literary,” “informal,” “archaic,” “vulgar slang,” “rare,” “dialect,” or “derogatory” would be less relevant to beginning learners. Similarly, any senses primarily used within specific domains such as “Physics” or “Law” are also disregarded. Such sense may be common in a large corpus which consists of mainly written texts for adults, but is less relevant to beginning learners. Moreover, words labeled as “proper nouns” are removed. Such words mainly include names of countries, cities or people such as “Britain,” “Rome,” or “Anne.” Terms signifying the language or inhabitants of these regions are also eliminated at this point. Last, senses primarily utilized in countries or areas outside of the UK or the US are dismissed as well. Although different regional dialects are equally important, the UK or the US variety is generally focused on first by young learners in foreign language education contexts.

Third, senses with a range below 0.5 are removed. Relying solely on frequency is unreliable especially when the variable does not follow a normal distribution (Gries, 2021). Thorndike (1932) might be the first to introduce the concept of distributional information in the development of word lists, specifically referring to how many sub-corpora or texts a word appears in at least once. However, this measure of distributional information is not too simple because it does not consider the size of the individual sub-corpora in which a word appears, nor does it take into account the frequency of occurrence of the word in the individual sub-corpora. To address this limitation, we draw from Gardner and Davies (2014) and make the following definition of distribution: if the frequency of a sense in a sub-corpus is ≥20% of its expected frequency, then the sense is said to be “distributed” in that sub-corpus; otherwise, it is not. COCA can be classified into eight sub-corpora based on genre: academic, blog, fiction, magazine, news, spoken, TV and movie, and web. If a sense is distributed in six out of the eight sub-corpora, its distributional score is calculated as 6/8 = 0.75. Although range is considered an important criterion in the development of word lists, no previous study has established a standardized reference for this factor. We choose to use 0.5 as the threshold after several pilot experiments. The standard can be refined in later studies.

Finally, we select the items that cover around 80% of COCA as high-frequency senses.

There is no consensus on the number of items that should be included in a high-frequency list. West's (1953) GSL consists of ~2,000 word families. This figure (2000) has long been utilized as a reference point for defining “high frequency” in the following studies. Nation (2001) also suggested that the 2,000 most frequent word families should be labeled as high-frequency vocabulary. However, the selection of 2,000 as the upper limit for high-frequency vocabulary lacks a specific rationale. Nation (2001) himself clearly stated that this decision is open to debate. Schmitt and Schmitt (2014) proposed that rather than using the traditional figure of the first 2,000 word families as the upper boundary of high-frequency vocabulary, the threshold should be moved and include the first 3,000 word families.

Instead of getting caught up in the debate of whether a high-frequency word list should consist of 2,000 or 3,000 items, we take a different approach. We aim to create a list that achieves a similar level of coverage as influential lists like GSL or New-GSL, which cover ~80% of different corpora. To achieve this, we will calculate the coverage of each set of 1,000 senses and select the number of items that can encompass around 80%.

### 3.5 Validity check

This high-frequency word list is derived from the COCA corpus, which represents contemporary American English. A natural concern is whether this list accurately represents other varieties of English, especially British English. To address this concern, we will check the coverage of this list in BNC, and further we will compare two lists created from an American English corpus and a British English corpus, respectively.

First, we will check the coverage of this list in the BNC corpus. Coverage refers to the percentage of words in a corpus that are represented by items from a specific word list (Nation and Waring, 1997; Dang et al., 2022). It is the primary criterion for evaluating word lists in previous studies, such as Nation (2004), Gilner and Morales (2008), and Brezina and Gablasova (2015). In this article, we will calculate the coverage of our high-frequency sense list in BNC, the only large corpus whose text can be downloaded and analyzed by BERT. If these high-frequency senses can provide a stable coverage in BNC as well as in COCA, we can infer that these senses are not biased toward American English.

A stable coverage, while an important indicator of being representative, is not sufficient. For example, if we create a high-frequency word list from BNC and it contains all the items found in the COCA list but in different ranking positions, the coverage in different corpora would be the same. However, the linguistic characteristics reflected by the two lists would be different. For instance, if on the BNC list Sense X is the first item and Sense Y is the last item, while on the COCA list, Sense Y is the first item and Sense X is the last, these two lists represent language characteristics despite having the same coverage in different corpora.

To address this issue, we will create two high-frequency lists from COCA and BNC respectively, and compare the ranking position of shared items in the lists, using Spearman's *rho* as a statistical measure.

To facilitate comparison, the spelling of American English words in COCA will be adjusted to align them with British spelling.^10^

## 4 Results and discussion

### 4.1 Semantic annotation

**Figure 2:**
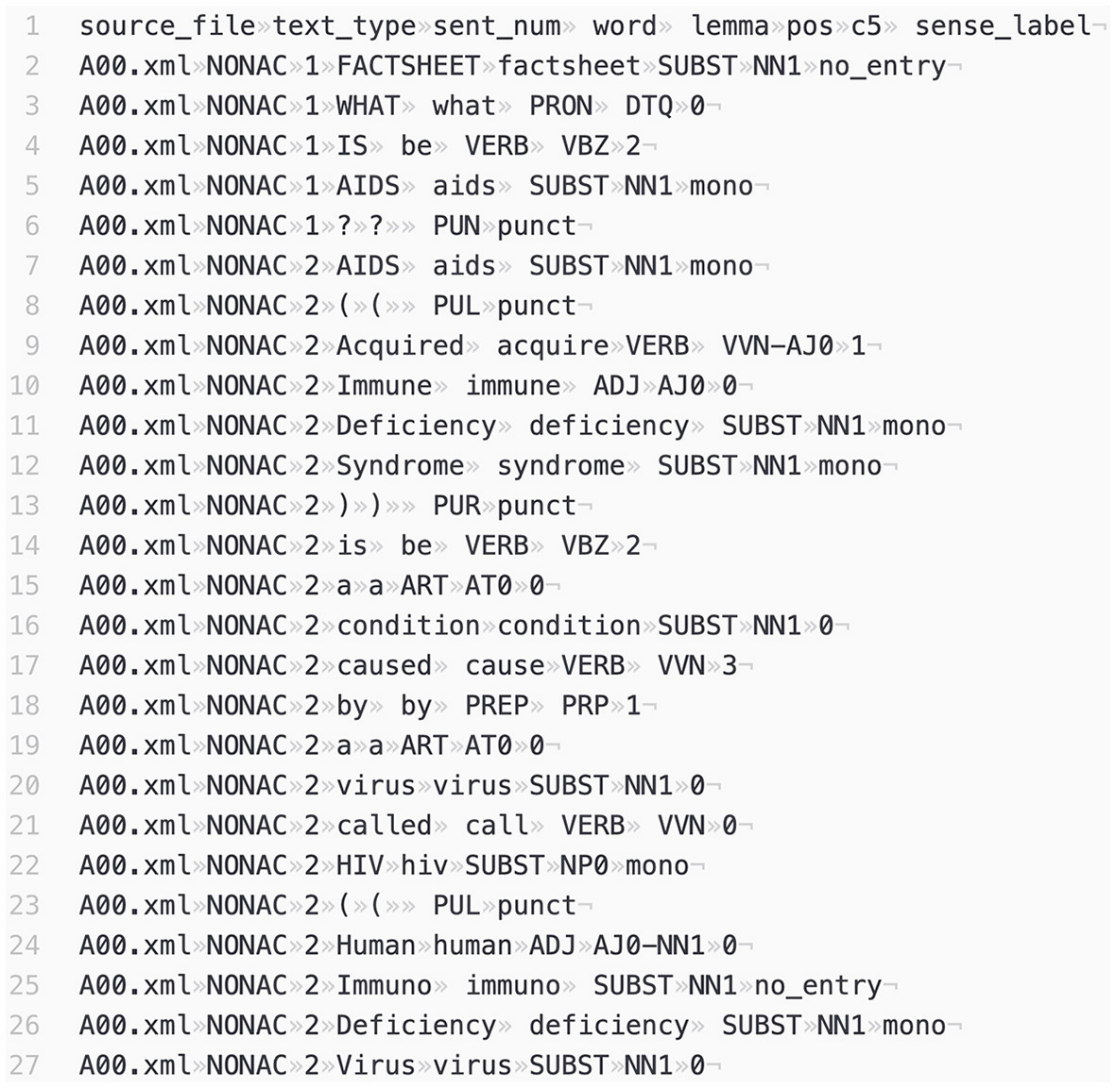
A snapshot of the semantically annotated COCA.

We annotated COCA using BERT, and Figure 2 is a snapshot of the annotated corpus. The annotated corpus specifies “source file,” “register,” “sentence No.,” “word type,” “lemma,” “pos,” “C5,” and “sense label.”

After the annotation, each word in COCA has a “sense label,” and each sense label matches a sense entry in the OED dictionary. As illustrated in Figure 2, the sense label assigned to “what” in the sentence “What is AIDS?” is “0.” This indicates that the meaning of “what” in this context corresponds to the first sense entry in the OED dictionary.^11^ Likewise, the meaning assigned to “cause” in the 17th line is labeled as “3,” indicating a match with the fourth sense entry in the OED.

### 4.2 Accuracy of annotation

After the annotation was completed, we evaluated the accuracy rate of BERT to disambiguate polysemous words.

The overall correct rate of BERT to disambiguate polysemous words is 94%. There are 63,365 word entries in OED. Out of these words, 19,653 words are polysemous. Of these polysemous words, 14,847 words have enough examples for at least two senses, as is shown in Table 1. Therefore, the annotation accuracy rate is based on 14,847 polysemous words that have a sufficient number of example sentences available.

**Table 1:** Different word types in OED.

**Word type**	**Sub-type**	**Word number**	**Sense number**	**Sentences checked**
Monosemy	Only one sense	43,712	43,712	142,709
Polysemy	No sense is illustrated with examples	3,473	8,457	0
	Only one sense is illustrated with examples	1,333	3,025	5,403
	At least two senses are illustrated with examples	14,847	46,694	272,798
Total		63,365	101,888	420,910

A total of 272,798 example sentences for these 14,847 words, after being annotated by BERT, are checked against the original definitions in OED. It is found that 257,285 sentences are correctly annotated. This results in an overall correct rate of 94%.

The accuracy rate in this study is higher than the results of previous sense disambiguation studies. In previous studies (Kilgarriff, 2001; Snyder and Palmer, 2004; Yuan et al., 2016), the inter-annotator agreement (IAA) typically falls within the range of 70–80%. It is noteworthy that even among human raters, achieving complete agreement on the exact meaning of a word is not consistently attainable.

It should be noted the rate of 94% indicates BERT's proficiency in distinguishing between multiple meanings of words. Considering that many words have only one meaning and are accurately annotated at 100%, the overall accuracy rate of COCA annotations is significantly higher.

### 4.3 High-frequency senses

Following the steps specified in Section 3.2, we selected high-frequency senses in COCA.

The overall distribution of sense frequency is found to adhere roughly to Zipf's law (Zipf, 1949; Nation, 2016). The first 1,000 senses encompass 65.35% of the total coverage, while the second 1,000 senses only contribute 7.39% of the coverage. The coverage gradually decreases with each successive set of 1,000 senses. After the seventh set, the coverage drops below 1%. It has been observed that the distribution of word frequency generally adheres to Zipf's law. A small number of words account for a significant portion of a given text (Nation, 2016). This pattern is also found in the distribution of sense frequency. The first 1000 high-frequency senses cover 65.35% in COCA, as is shown in Table 2.

**Table 2:** The coverage of each 1,000 senses in COCA.

**n-th 1,000**	**Coverage (%)**	**Cumulative coverage (%)**
1	65.35	65.35
2	7.39	72.74
3	4.16	76.89
4	2.72	79.61
5	1.96	81.57

Similar to previous findings of word frequency studies, the most commonly occurring items are generally grammatical words. Table 3 shows the first 20 most frequent senses in COCA.^12^

**Table 3:** Examples of high-frequency senses.

**Rank**	**Lemma**	**Pos**	**Sense definition**	**Norm freq**
1	Be	Auxiliary verb	Used with a present participle to form continuous tenses.	30,661.48
2	The	Determiner	Denoting one or more people or things already mentioned or assumed to be common knowledge.	20,833.57
3	A	Determiner	Used when referring to someone or something for the first time in a text or conversation.	19,072.07
4	And	Conjunction	Used to connect words of the same part of speech, clauses, or sentences, that are to be taken jointly.	14,297.49
5	To	Infinitive particle	Used with the base form of a verb to indicate that the verb is in the infinitive.	12,970.09
6	I	Pronoun	Used by a speaker to refer to himself or herself.	12,899.41
7	Of	Preposition	Following a noun derived from or related to a verb.	10,738.16
8	You	Pronoun	Used to refer to the person or people that the speaker is addressing.	6,836.45
9	That	Conjunction	Introducing a subordinate clause expressing a statement or hypothesis.	6,777.34
10	He	Pronoun	Used to refer to a man, boy, or male animal previously mentioned or easily identified.	5,623.24
11	It	Pronoun	Used to refer to a thing previously mentioned or easily identified.	5,118.69
12	Have	Verb	Possess, own, or hold.	4,855.55
13	And	Conjunction	Used to introduce an additional comment or interjection.	4,586.23
14	In	Preposition	Expressing the situation of something that is or appears to be enclosed or surrounded by something else.	3,990.29
15	You	Pronoun	Used to refer to any person in general.	3,912.32
16	Of	Preposition	Expressing the relationship between a general category or type and the thing being specified which belongs to such a category.	3,893.89
17	They	Pronoun	Used to refer to two or more people or things previously mentioned or easily identified.	3,793.5
18	We	Pronoun	Used by a speaker to refer to himself or herself and one or more other people considered together.	3,786.81
19	The	Determiner	Used to make a generalized reference to something rather than identifying a particular instance.	3,637.45
20	Say	Verb	Utter words so as to convey information, an opinion, a feeling or intention, or an instruction.	3,494.02

Each word in the list represents a single specific meaning, rather than serving as an umbrella term for multiple meanings. For instance, the word “to,” which ranks 5th in this list, refers exclusively to its infinitive particle usage as shown in example ④ and does not encompass its prepositional meaning as illustrated in ⑤.

④ I set out to buy food.
⑤ He is married to Emma.

We selected the first 5,000 senses as high-frequency senses and created a high-frequency sense list (HFSL). We set the cut-off point at 5,000 because these senses offer coverage similar to that of previous word lists in large corpora. GSL, NGSL and New-GSL cover ~80% of various corpora such as BNC, BE06, and EnTenTen12. Similarly, the first 5,000 senses we selected cover 81.57% of COCA.

To compare the coverage of different word lists, we first standardized the spelling system. The words in GSL and New-GSL use British English norms, while words in NGSL use American English norms. We converted all the spellings into British English when checking coverage in British English corpora such as BNC, and into American English when checking coverage in American English corpora such as COCA.

Following this spelling standardization, we created a sense list for GSL, NGSL, and New-GSL. Different word lists use different counting units. GSL consists of ~2,000 word families, NGSL consists of 2,809 flemmas, while New-GSL consists of 2,494 lemmas. To make a valid comparison, we counted the respective number of senses in these lists in OED. The 2,000 words from GSL collectively have 12,616 senses; NGSL has 11,525 senses, and New-GSL has 6,971 senses.

After these standardization steps, we compared the number of senses in different word lists. The results reveal that comparable coverage is achieved with significantly fewer items. As indicated in Table 4, the HFSL achieves similar coverage, around 80%, with only half the senses of GSL or NGSL, or 70% of the senses of New-GSL.

**Table 4:** Comparison of the coverage of GSL, New-GSL, and HFSL.

	**Word family**	**Flemma**	**Lemma**	**Sense**	**Coverage in COCA (%)**
GSL	2,000		4,114	12,616	79.25
NGSL		2,809		11,525	80.12
New-GSL			2,494	6,971	78.49
HFSL		3,621	3,179	5,000	81.57

As we transition from larger units like word families to smaller units like flemmas or lemmas, and finally to senses, we can achieve comparable coverage with a reduced number of items. In essence, by using senses as the counting unit, we can select high-frequency items more accurately.

HFSL contains more items than NGSL or New-GSL in terms of lemma or flemma. When the 5,000 senses are converted to lemmas or flemmas, there are 3,621 flemmas or 3,179 lemmas in HFSL. Nonetheless, this aligns with the process of vocabulary acquisition. Language learners typically don't learn all the senses of a specific word before moving on to the next. They often first acquire the common senses of a word and then proceed to learn a new word.

### 4.4 Validity of HFSL

The high-frequency list is generally representative of British English, because this list achieves a similar coverage in BNC, and moreover, items in two lists created from BNC and COCA respectively are similar in ranking positions.

First, these high-frequency senses have a similar coverage in BNC. The coverage of these 5,000 high-frequency senses in BNC is 77.47%. It is a bit lower than the coverage in COCA (81.57%), but it is still relatively stable. This means that most high-frequency senses are shared by American English and British English.

Second, the two lists created from BNC and COCA are similar in terms of both shared items and ranking positions. We created two lists of high-frequency senses from COCA and BNC respectively, and then compared the first 5,000 items in two lists. It is found that 81% of the items in the two lists are identical. What is more important, the ranking position of the common items in both lists is similar. The correlation between the common items in both lists was determined using Spearman's *rho* (Oakes, 1998). The correlation coefficient was found to be 0.82, indicating a strong positive correlation (*p* < 0.001). This suggests that the ranking position of common items in both lists is similar.

This indicates that high-frequency senses have cross-regional stability, with no significant variations in the two major English varieties, British English and American English. This echoes Dang and Webb's (2016) finding that high-frequency words achieve similar coverage in different regional varieties of English.

Therefore, a list based on American English can serve as a useful resource, at least as a useful starting point, for foreign language learners aiming to understand English in general.

### 4.5 Pedagogical and methodological implication

Creating such a high-frequency sense list has both pedagogical and methodological implications. Pedagogically, it allows us to confidently suggest which senses should be prioritized by beginning learners. Methodologically, it opens up a new approach to accurately identifying high-frequency items.

The list indicates which senses are more frequently used and therefore should be focused on in foreign language education. Table 5 presents the semantic frequency of different senses of the word “act,” from which we can safely say that “act^1^” (the written law) is more frequently used than other senses, while “act^8^” (pretense) is less frequently used.

**Table 5:** Semantic frequency of the lemma “act.”

**Sense no**.	**Pos**	**Sense definition**	**Example**	**Frequency in COCA**
1	Noun	A written law passed by Parliament, Congress, etc.	The 1989 Children Act	45,360
2	Noun	A thing done; a deed.	A criminal act	41,691
3	Verb	Take action; do something.	They urged Washington to act.	30,671
4	Verb	Behave in the way specified.	They challenged a man who was seen acting suspiciously	29,803
5	Verb	Perform a role in a play, film, or television.	She acted in her first professional role at the age of six	16,224
6	Verb	Take effect; have a particular effect.	Blood samples are analyzed to find out how the drug acts in the body	14,484
7	Noun	A main division of a play, ballet, or opera.	The first act	13,873
8	Noun	A pretense.	She was putting on an act and laughing a lot.	4,678

This has great pedagogical implications. As previous word lists failed to describe which senses are frequent and which are less widely used. Classroom teachers and curriculum designers have had to rely on their expertise to determine which meanings should be prioritized initially. However, it is not always reliable. For example, the word list in the *Shanghai Guideline for English Teaching in Middle School* (“*Shanghai Teaching Guidelineline*”) includes some less frequent senses while excluding lots of high-frequency senses.

In *Shanghai Teaching Guideline* some less frequent senses are required to be mastered in middle school. Some typical examples include the “intelligent” sense of “able,” the verbal use of “pain,” and the “pulling” sense of “draw.” These words—“able,” “pain,” and “draw”—are undoubtedly common, but these specified meanings mentioned above are not common. For example, despite “able” is a commonly used word, its “intelligence” sense is not frequently used. In COCA, there are 214,842 occurrences of “able” with the sense of “having the power, skill, means, or opportunity to do something.” In contrast, there are only 4,951 occurrences of its “intelligent” sense. Similarly, “pain” is a common word, with over 80,000 occurrences in COCA, but its verbal sense is infrequent, appearing only 1,110 times. It is recognized that frequency alone does not determine which words or senses should be prioritized. West (1953) points out that factors such as ease of difficulty in learning, necessity in communication, and stylistic impact all play significant roles. However, the mentioned senses do not meet these standards. The “intelligent” sense of “able” can easily be substituted by more common words, and the verbal use of “pain” is primarily found in literature.

**Figure 3:**
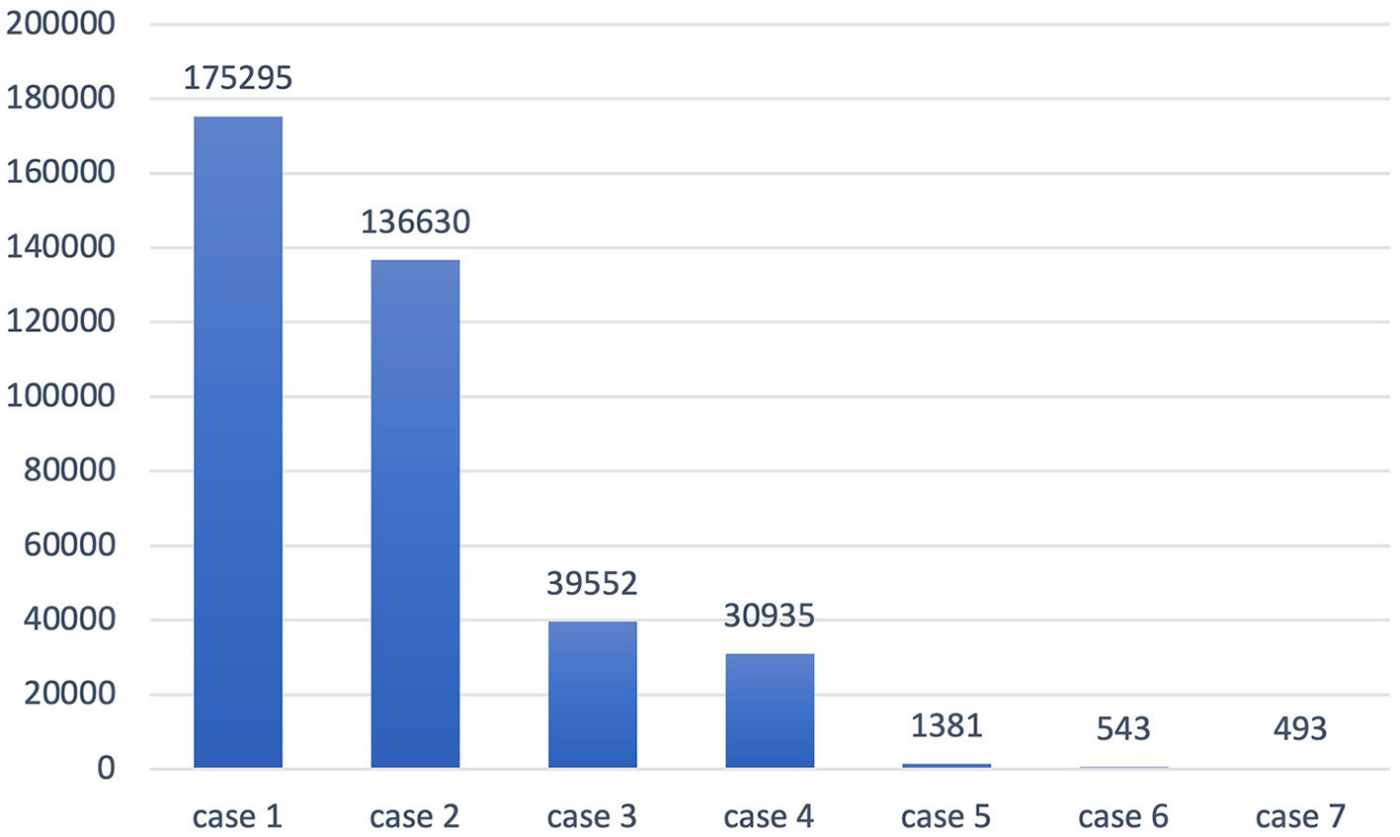
Frequency of different senses of the word “case.”

On the other hand, a lot of high-frequency senses are omitted in the *Shanghai Teaching Guideline*. For instance, the word “case” has seven senses in OED, as is shown in Table 6. These senses have different frequencies in COCA, as exhibited in Figure 3. The first meaning is most frequently used, occurring 175,295 times, but it is not specified in the *Shanghai Teaching Guideline*, where the word “case” only has two meanings, the legal action sense (case^2^) and the container meaning (case^4^).

**Table 6:** Definition of “case” in OED.

**No**.	**Definition**
Case^1^	An instance of a particular situation; an example of something occurring.
Case^2^	A legal action, especially one to be decided in a court of law.
Case^3^	An instance of a disease, injury, or problem.
Case^4^	A container designed to hold or protect something.
Case^5^	Surround in a material or substance.
Case^6^	Each of the two forms, capital or minuscule, in which a letter of the alphabet may be written or printed.
Case^7^	Any of the forms of a noun, adjective, or pronoun that express the semantic relation of the word to other words in the sentence.

More examples can be given easily. “Certain” can mean either “able to be firmly relied on to happen or be the case” or “specific but not explicitly named or stated;” “adopt” can mean either “legally take (another person's child) and bring it up as one's own” or “choose to take up, follow, or use;” “base” can mean either “a place used as a center of operations by the armed forces or others” or “use (something specified) as the foundation or starting point for something.” Each sense is illustrated with the following examples.

⑥There are many people eager to adopt a baby (adopt^1^).
⑦This approach has been adopted by many big banks (adopt^2^).
⑧He headed back to base (base^1^).
⑨The film is based on a novel by Pat (base^2^).
⑩I'm not certain who was there (certain^1^).
⑪ The museum is only open at certain times of the day (certain^2^).

**Figure 4:**
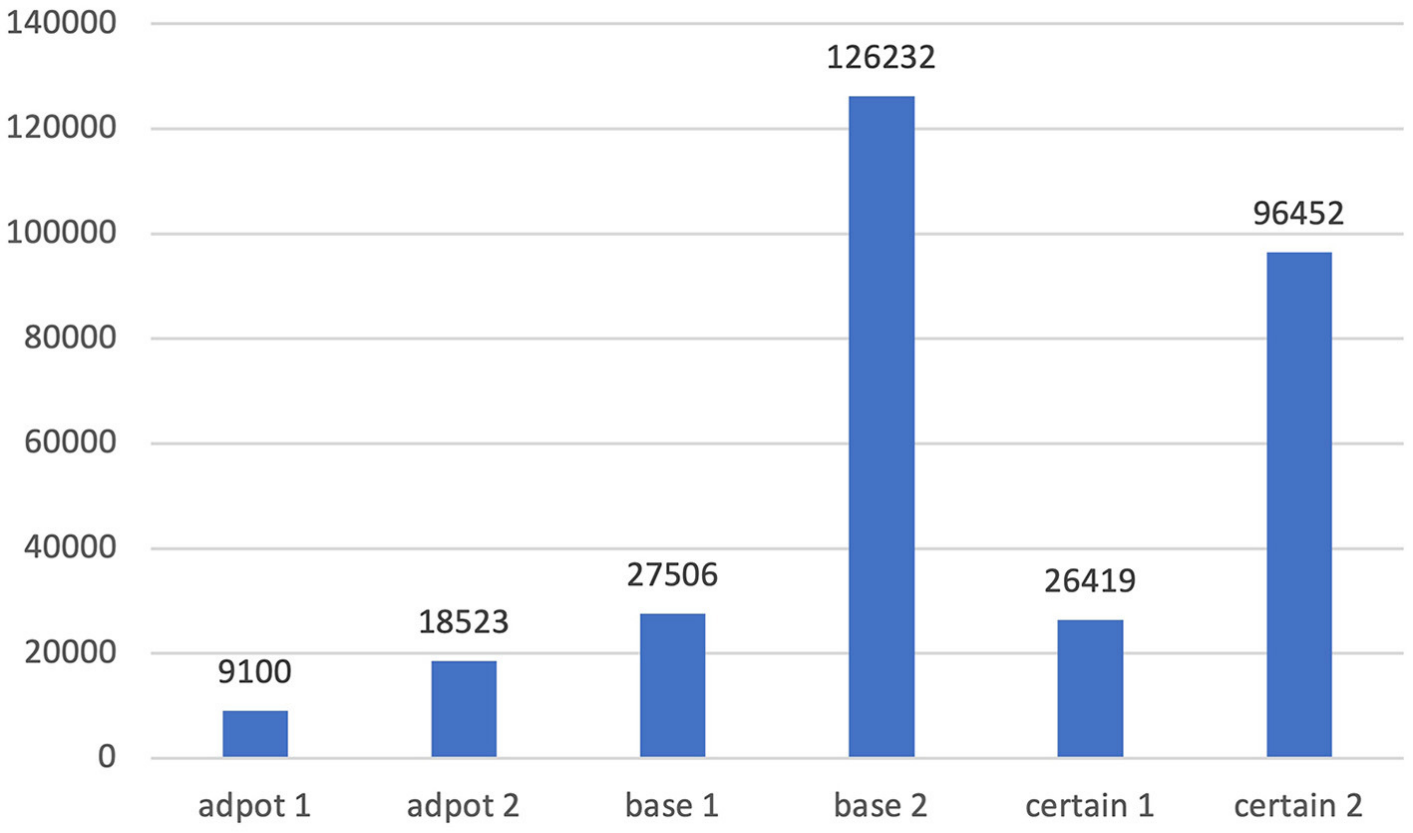
Frequency comparison of different senses of “adopt,” “base,” and “certain.”

As is shown in Figure 4, the second meaning in each pair is much more frequent in COCA. However, in the *Shanghai Teaching Guideline*, only the first meaning is provided. That is to say, the most frequent sense is omitted.

Another systematic omission in the *Shanghai Teaching Guideline* is the neglect of high-frequency metaphorical meanings. The word “beat” has a metaphorical meaning (example ⑫) as well as a literal meaning (example ⑬). Statistics of COCA show that the metaphorical meanings are much more frequently used. However, only the literal meaning is listed in the *Shanghai Teaching Guideline*. Similar examples include “aim,” “circle,” “degree,” “argue,” “direction,” etc.

⑫ Our team beat Germany 3-1.
⑬ She beat the dog with a stick.

The neglect of metaphorical meanings can also be observed in such words as “deep,” “large,” and “heavy.” Their metaphorical meanings are used at least as frequently as their literal meanings in COCA. In the case of “deep,” there are 45,382 occurrences of its metaphorical meaning, twice the occurrences of its literal meaning.

⑭ She was in deep trouble.
⑮ A deep gorge.

Metaphorical meanings have generally been excluded in previous foreign language curricula because they are thought to be hard for children to understand. It is indeed true that children acquire concrete meanings first before they learn and use them metaphorically. However, this does not entail that middle school students in foreign language contexts have trouble learning these meanings. As Laufer (1997) pointed out, foreign language learners in middle school have already developed abstract thinking and are capable of understanding metaphorical meanings.

To sum up the pedagogical implications, the high-frequency sense list created in this study can help teachers and learners understand clearly which senses should be mastered first.

Methodologically, this study changed the counting unit in lexical frequency studies from word family or lemma to senses. By narrowing the counting unit, we can identify high-frequency items accurately.

This list includes common words without requiring any additional adjustment. When creating the BNC/COCA list, Nation found that many common words, such as days of the week cannot enter the high-frequency band solely based on frequency. The problem is similarly faced by Brezina and Gablasova (2015). In New-GSL, common words such as “apple” and “tiger” did not get into the list.

The concept of “word” in previous studies represents a number of distinct or even unrelated meanings. Naturally, words of only one or a few senses such as “Monday” do not have more occurrences than those words that have a large number of senses. To solve this problem, a spelling form should be matched with a single meaning. This has never been achieved in previous studies due to technical constraints (Gardner, 2007; Gardner and Davies, 2007). The recent advancement of large language models presents an opportunity to bridge this gap. In this study, we, using BERT, annotated large English corpora semantically, which is an aim that has long been expected to achieve.

After the semantic annotation of COCA, we counted the frequency of different senses. This method automatically includes words with single meanings in the high-frequency range. As illustrated in Table 7, words such as “Monday” or “April” attain a high position in the list.

**Table 7:** Ranks of common words.

**Word**	**Ranking position in COCA**
Monday	1,541
Thursday	1,856
April	1,434
Tiger (the large cat sense)	3,939
Apple (the fruit sense)	2,351
Hungry	3,485

## 5 Conclusion

In this article, we use BERT to annotate COCA semantically and create a high-frequency list of senses. This list boasts a comparable coverage as GSL, NGSL, or New-GSL, but with a significantly reduced number of items. Despite being sourced from COCA, this list represents both American English and British English. It has stable coverage in BNC; it exhibits similar ranking positions with a list created from BNC.

By creating such as list, we can explicitly identify the frequently used senses of polysemous words. Such a word list, complete with semantic frequency information, better meets the needs of both teachers and students compared to previous word lists. Furthermore, by counting senses rather than word families or lemmas, we can more precisely identify high-frequency items.

This list can serve as a starting point for developing word lists. Although we are aware that frequency is not the sole criterion for creating an ideal word list. Additional factors such as psychological reality are also important. In children's eyes, high-frequency words such as “government” or “president” are not necessarily more important than less frequent words such as “tiger” or “bear.” Similarly, these high-frequency senses are not necessarily the earliest usage acquired by young learners. In children's life, act^3^, act^4^, and act^5^ might be more important. Children will learn these senses first. Similarly, children might acquire the literal meaning of “deep” first before the metaphorical meaning. However, these high-frequency senses still deserve serious attention, as they are used widely in future life. This list can serve as a starting point for further refinement.

A limitation of this study is we only annotated single words and phrasal verbs. We did not treat longer language units such as idioms and sayings separately. This will be addressed in the future when the capacity of large language models continues to advance.

## Data availability statement

The datasets presented in this article are not readily available because they can be used for academic purpose only. Requests to access the datasets should be directed to the corresponding author.

## Author contributions

TG: Writing – original draft. LL: Data curation, Writing – original draft. JS: Conceptualization, Writing – review & editing. YG: Validation, Writing – review & editing.

## Footnotes

^1^The lists developed by Browne et al. (2013) and Brezina and Gablasova (2015) are both referred to as the New General Service List. The former is commonly abbreviated as NGSL, while the latter is New-GSL.

^2^Also refer to the introduction of NGSL on its website. https://www.newgeneralservicelist.com/new-general-service-list.

^3^The NGSL supplementary words can be found at: https://www.newgeneralservicelist.com/new-general-service-list.

^4^We are grateful for the Research License provided by Oxford Dictionaries. https://developer.oxforddictionaries.com, retrieved on 2022-10-2.

^5^Many phrasal verbs also have multiple meanings. Our attempts to accurately distinguish between these different meanings using the current BERT model have been unsuccessful. In this study, the various meanings of a phrasal verb are treated as a single entity. This issue will be refined in later studies.

^6^ https://www.english-corpora.org/coca/

^7^ http://www.natcorp.ox.ac.uk/XMLedition/index.xml?ID=intro

^8^ https://github.com/google-research/bert

^9^We do not distinguish homonymy from polysemy in this study.

^10^ http://heiswayi.github.io/spelling-uk-vs-us/

^11^“0” represents the first sense entry, “1” represents the second sense entry, and so forth in the OED dictionary.

^12^Due to space limitations, we have omitted certain columns in this table, such as “sub-sense,” “example sentence,” “register,” “domain,” etc.
